# Cord Blood 25-Hydroxyvitamin D Level is Correlated with a Risk for Atopic Dermatitis: Systematic Review and Meta-Analysis

**DOI:** 10.21315/mjms2024.31.4.4

**Published:** 2024-08-27

**Authors:** Dedianto Hidajat, Abiyyu Didar Haq, Cut Warnaini, Hamsu Kadriyan

**Affiliations:** 1 Department of Dermatology and Venereology, Faculty of Medicine, University of Mataram, Indonesia; 2 Medical Doctor Profession Program, Faculty of Medicine, University of Mataram, Indonesia; 3 Department of Public Health, Faculty of Medicine, University of Mataram, Indonesia; 4 Department of Otolaryngology, Head and Neck Surgery Faculty of Medicine University of Mataram, Indonesia

**Keywords:** cord blood, 25[OH]D deficiency, atopic dermatitis

## Abstract

Although Indonesia is located in an equatorial region with adequate year-round sun exposure, the prevalence of 25-hydroxyvitamin D (25[OH]D) deficiency is as high as 90%. Mothers are especially vulnerable to deficiencies due to changes in their gastrointestinal system. Previous studies have reported a correlation between the 25[OH]D status of mothers with atopic dermatitis (AD) and their offspring. However, studies investigating maternal cord blood 25[OH]D levels and the incidence of AD have yielded controversial results due to its variability. As such, this systematic review and meta-analysis aimed to evaluate the correlation between maternal cord blood 25[OH]D levels and the risk for AD. In accordance with Preferred Reporting System for Systematic Reviews and Meta-Analyses (PRISMA) guidelines, the PubMed, Cochrane Library and ScienceDirect databases were searched for relevant observational studies and a meta-analysis was performed to obtain odds ratios (OR) and corresponding 95% confidence intervals (CI). Nine studies were included in the qualitative synthesis, five of which were included in the quantitative synthesis. Meta-analysis revealed that cord blood 25[OH]D levels < 50 nmol/L were associated with a 60% higher risk for the development of AD (OR = 1.60; 95% CI: 1.15, 2.22; I^2^ = 0%; *P* < 0.05). However, qualitative synthesis revealed a variety of cord blood 25[OH]D measurements and different methods of diagnosing AD in each study. Based on the current analysis, maternal cord blood 25[OH]D levels were significantly correlated with the risk for AD. Therefore, studies investigating 25[OH]D supplementation in pregnant women and its efficacy in decreasing the risk for AD are needed, especially in tropical and equatorial countries. This study also serves as a proof of concept that cord blood 25[OH]D levels can be used as a more affordable predictive parameter for AD.

## Introduction

Vitamin D (25-hydroxyvitamin D (25[OH]D) plays a key role in innate and adaptive immunity by stimulating various receptors, such as toll-like receptors, increasing the production of proinflammatory cytokines and increasing the response of type 2 T-helper cells. This mechanism is the basis of many studies investigating the correlation between 25[OH]D levels and the incidence of allergy (1). Vitamin D deficiency is defined as a serum level < 50 nmol/mL (2). The main source of 25[OH]D in humans is the synthesis of 7-dehydrocholesterol in the skin upon exposure to ultraviolet-B light, which is converted into a detectable and measurable metabolite, 25[OH]D (3, 4). Circulating 25[OH]D level is a widely used parameter for 25[OH]D sufficiency (5).

Low 25[OH]D intake and lack of sunlight exposure are the main causes of deficiency (6). Several other factors, including body mass index (BMI), skin pigmentation, geographical characteristics, type of clothing, use of sunscreen, intensity of outdoor activities, and genetic and age, affect the synthesis and intake of 25[OH]D, especially among pregnant women (7–9).

Throughout pregnancy, the foetus is able to fulfill 25[OH]D needs from cord blood supply and the ability of 25[OH]D to cross the placenta (10, 11). Indonesia, a country located in the equatorial region, has adequate year-round sun exposure. This fact contradicts a recent study that reported the prevalence of vitamin D deficiency is as high as 90% (12, 13).

Currently, allergy has increasing prevalence among infants and children (14–16). One of these is atopic dermatitis (AD), which occurs more often in infants, indicating a correlation with early phases of life (17, 18). The International Study of Asthma and Allergies in Childhood (ISAAC) reported that the prevalence of AD is 15%–20% among infants and children, while only 1%–3% in adults (19, 20). Although its cause is multifactorial, various studies have found that 25[OH]D deficiency during pregnancy has a negative effect on the development of the immune system in the offspring (21, 22). Allergen sensitisation is the main risk factor for atopic diseases. The incidence and severity of atopic diseases have been confirmed to be directly related to allergen sensitisation during the early phase of life, which further supports the hypothesis of a correlation between maternal 25[OH]D deficiency and the incidence of allergic diseases in children (23–25).

Several published studies have demonstrated a correlation between maternal 25[OH]D status and the incidence of AD. However, studies regarding maternal cord blood vitamin D levels and the incidence of AD have yielded controversial results due to its high variability, indicating the need for pooled results from all studies addressing this health this matter. Currently, cord blood samples are mainly collected for blood gas measurements and stem cell banking (26–28). Therefore, this systematic review and meta-analysis aimed to evaluate the correlation between maternal cord blood 25[OH]D levels and risk for AD.

## Methods

### Search Strategy

This systematic review of clinical trials was conducted in accordance with the Preferred Reporting System for Systematic Reviews and Meta-Analyses (PRISMA) guidelines. A literature search of the PubMed, ScienceDirect, Cochrane Controlled Register of Trials (Central) and Wiley databases for relevant studies published up to 10 July 2022, was performed using the following keywords or terms: ‘(25-hydroxyvitamin D (25[OH]D) OR 25-hydroxy 25-hydroxyvitamin D (25[OH]D)) AND (Maternal Cord blood OR Cord blood) AND Atopic Dermatitis.’

### Inclusion and Exclusion Criteria

**Figure 1:**
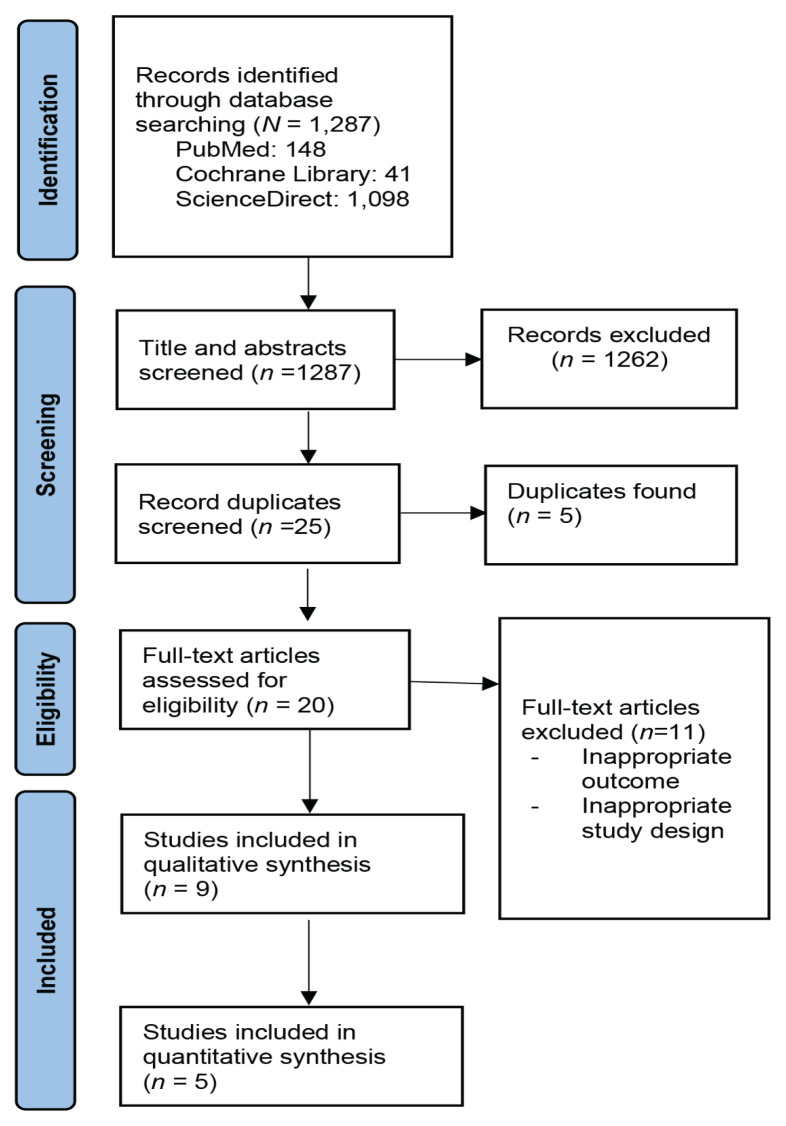
Diagram flow of literature search strategy for this systematic review and meta-analysis (29)

Studies investigating the correlation between cord blood 25[OH]D levels and the risk for AD using an observational design were included. Studies with irretrievable full-text articles and those published before 2000 were excluded. Details of the study search strategy are presented in Figure 1.

### Data Extraction and Quality Assessment

Data from the selected articles were extracted, including the following: author and year of publication; sample characteristics and size; assessment methods; and primary outcome of the incidence of AD. The studies were also assessed for quality according to the Strengthening the Reporting of Observational Studies in Epidemiology (STROBE) criteria. The checklist consists of 22 criteria, each scored 1 point, with a total maximum score of 22 points. Quality assessment was performed collaboratively by all the reviewers until consensus was reached. Results of the risk of bias analysis are presented in Appendix.

### Statistical Analysis

The meta-analysis was performed using Review Manager version 5.4 (Copenhagen, Nordic Cochrane Center, Cochrane Collaboration). Quantitative synthesis was performed using inverse variance methods with the DerSimonian Laird random-effects model because moderate to high heterogeneity was anticipated in the included studies (30). Odds ratio (OR) and corresponding 95% confidence interval (CI) was selected as the common measure of the correlation between maternal cord blood 25[OH]D levels and the incidence of AD. Differences with *P* < 0.05 were considered to be statistically significant. The Higgins I-squared (I^2^) statistic model was used to measure heterogeneity of the pooled results. Heterogeneity was classified as follows: negligible (I^2^ = 0%–24%); low (I^2^ = 25%–49%); moderate (I^2^ = 50%–74%); and high (I^2^ > 75%) (31).

## Results

### Study Selection

The initial search of all databases yielded 1,287 studies, of which 1,262 were excluded after screening titles and abstracts. Additionally, five were duplicates and, therefore, excluded. Subsequently, 11 additional studies were excluded because their outcomes were not relevant to this review. Ultimately, nine clinical trials were included for the qualitative analysis, five for the quantitative analysis and all were observational cohort studies.

### Study Characteristics and Quality Assessment

The main characteristics of the included studies in this systematic review are summarised in Table 1. A total of 3,952 mother-child pairs were enrolled, comprising studies published between 2012 and 2017. All of the studies were cohort studies, most of which were conducted in Australia and Europe.

**Figure 2:**
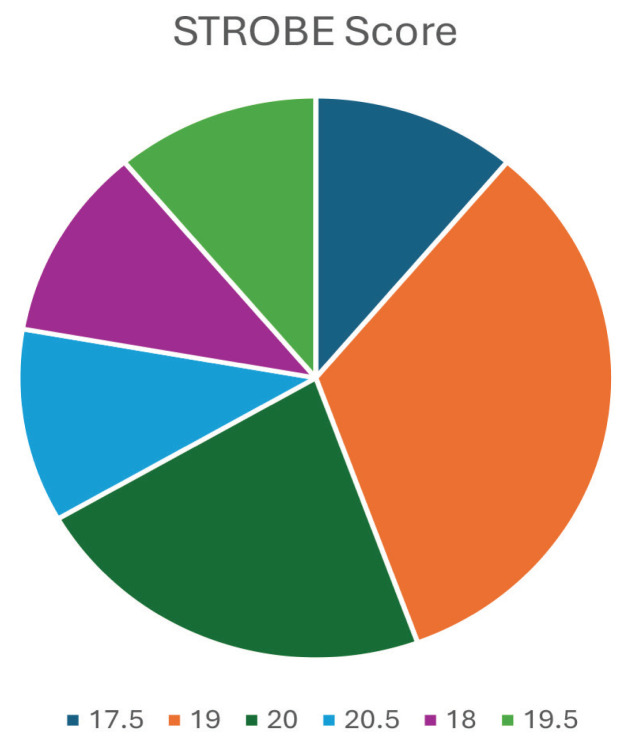
Graphical presentation of risk assessment result of the included studies using STROBE’s criteria of cohort studies

In terms of risk assessment, of all included studies, the lowest calculated STROBE score was 17.50/22.00 (range 17.50–20.50), which are graphically presented in Figure 2. This means that in all studies, more than two-thirds of the criteria were fulfilled (> 14.67/22.00), indicating that all included studies had a lower risk of bias and were of relatively good quality.

## Discussion

### Correlation between Maternal Cord Blood 25[OH]D Level and the Risk for AD

**Figure 3:**
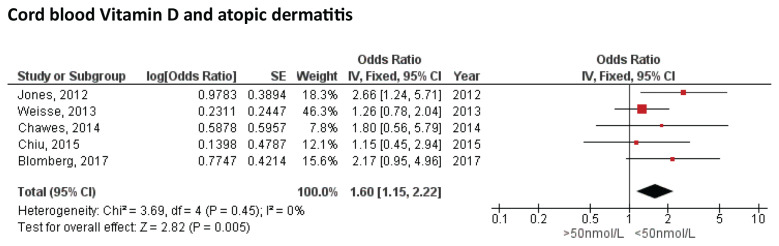
Pooled result of the correlation between low cord blood 25[OH]D level with the risk of atopic dermatitis

Five studies reported a correlation between 25[OH]D levels and the risk for AD (32–36). All studies demonstrated that maternal cord blood 25[OH]D levels < 50 nmol/L were associated with a greater risk for developing AD, although with variable results. The highest risk was reported by Jones et al. (32) in 2012 with an OR of 2.66 (95% CI: 1.24, 5.71), which included healthy pregnant women who underwent full-term delivery without complications. This study observed AD in infants 12 months of age, whose parents had a history of allergic diseases. On the other hand, the lowest risk was reported by Chiu et al. (32) in 2015 (OR = 1.15; 95% CI: 0.45, 2.94), which included a similar sample characteristic as Jones et al. This contradictory result was believed to be caused by the different inclusion criteria, specifically in terms of the history of allergic disease in the parents. In the study by Jones et al. (37), one of the inclusion criteria was a history of allergic disease in at least one of the offspring’s parents, whereas in the study by Chiu et al. (23), the aforementioned history was an exclusion criterion. Weisse et al. (35) used a history of allergic disease as an exclusion criterion. They reported that a history of allergic disease in parents was a confounding factor for the risk of AD in the offspring. Our meta-analysis (Figure 3) revealed that a cord 25[OH]D level < 50 nmol/L was associated with a 60% higher risk for the development of AD (OR = 1.60; 95% CI: 1.15, 2.22]; I^2^ = 0%; *P* < 0.05).

This result can be explained by several biological pathways that include the involvement of 25[OH]D in the pathogenesis of AD, which directly correlates with immune dysregulation, epidermal defense disturbances and inadequate bacterial defense(s) (38). It has also been reported that a higher 25[OH]D concentration in cord blood exhibits a direct correlation with a decreased risk for allergic disease mediated by immunoglobulin (Ig) E, even though further study is urgently required (39).

Our results were similar to those of a previous study that reported a correlation between lower maternal vitamin D status during pregnancy and an increased risk for childhood eczema (40). A systematic review and meta-analysis revealed a modest association between low maternal vitamin D levels and an increased risk for childhood eczema in offspring. Another study reported no significant differences in the risk for AD between deficient and sufficient mothers, indicating a high variety or confidence interval in that particular method of correlation assessment (41). Another recent cohort study highlighted a possible mechanism underlying the association between the level of vitamin D during pregnancy and the risk for atopic dermatitis through the downregulation of FOXP3 gene expression in the cord blood and decreased placental FOXP3 protein expression. Low placental FOXP3 protein levels are related to activation of the PI3K/AKT/mTOR signaling pathway (42). This study found a positive correlation between maternal 25(OH)D3 levels and FOXP3 expression in the cord blood. Compared to women with vitamin D sufficiency, placental FOXP3 protein expression was decreased and PI3K/AKT/mTOR protein was upregulated. Zeng et al. (43) found an association between higher vitamin D levels in cord blood and a reduced risk for eczema in cohort studies. Therefore, this study proposes a better method for assessing the correlation between vitamin D levels and the risk for AD through cord blood 25[OH]D measurement.

The current investigation, however, had several limitations. First, the included studies used a variety of cord blood 25[OH]D measurement methods, which potentially increased the risk of bias. Therefore, we suggest that further studies comparing these measurement methods be conducted to improve the measurement method across all studies investigating this particular topic. Second, most of the included studies used various diagnostic methods for AD. Future studies should develop refined and consolidated diagnostic criteria based on our current understanding of the disease, as suggested by the self-report questionnaire that was used in most of our included studies (44–46).

## Conclusion

Based on the current analysis, maternal cord blood 25[OH]D levels were significantly correlated with the risk for AD. Therefore, studies investigating supplementation of 25[OH]D in pregnant women and its efficacy in decreasing the risk for AD are needed, especially in tropical and equatorial countries where sun exposure is adequate and the prevalence of vitamin D deficiency remains high (12). This study also serves as a proof of concept that cord blood 25[OH]D measurement can be used as a more affordable predictive parameter for AD.

## Figures and Tables

**Table 1:** Characteristic of the included studies

Author, Year	Study location	Study design	Population	Cord blood 25[OH]D measurement	Atopic dermatitis outcome	Follow-up duration (child)	Mother’s age range	OR (95% CI)	OR (95% CI) [every 25 nmol/L increase]	STROBE assessment result
Jones et al. (32) 2012	Australia	Cohort	231	Liquid chromatography-tandem mass spectometry (LC-MS/MS)	Doctor’s diagnosis and SCORAD	1 year	27.9–37.9	2.66 (1.24, 5.71)		79, 5%
Baiz et al. (46) 2014	France	Cohort	239	Immunochemiluminescent immunoassay	Doctor’s diagnosis and ISAAC questionnaire	5 years	26.2–35.4	n/a	0.87 (0.77, 0.98)	
Weisse et al. (35) 2013	Germany	Cohort	378	High performance liquid chromatography-tandem mass spectroscopy (HPLC-MS/MS)	Doctor’s diagnosis	2 years	n/a	1.26 (0.78, 2.02)	n/a	
Chawes et al. (34) 2014	Denmark	Cohort	257	LC-MS/MS	Hanifin-Rajka criteria	7 years	20.9–41.1	1.80 (0.56, 5.79)	n/a	93, 2%
Chiu et al. (33) 2014	Taiwan	Cohort	258	Electrochemiluminescence-based assay	ISAAC questionnaire and Hanifin-Rajka criteria	4 years	n/a	1.15 (0.45, 2.94)	n/a	
Stelmach et al. (45) 2015	Poland	Cohort	240	High performance liquid chromatography (HPLC)	ISAAC questionnaire	2 years	25.8–33.6	n/a	0.97 (0.86, 1.09)	
Jones et al. (37) 2015	Australia	Cohort	225	LC-MS/MS	ISAAC questionnaire and SCORAD	2.5 years	28.5–37.5	n/a	0.84 (0.72, 0.98)	
Palmer et al. (38) 2015	Australia	Cohort	706	LC-MS/MS	Hanifin-Rajka criteria	3 years	23.2–34.4	n/a	0.89 (0.81, 0.98)	
Blomberg et al. (36) 2017	USA	Cohort	1,418	High performance liquid chromatography (HPLC)	ISAAC questionnaire	4.5 years	n/a	2.17 (0.90, 5.21)	n/a	

## Appendix

### PRISMA 2020 main checklist

[Table t2-04mjms3104_ra]

**Table t2-04mjms3104_ra:**

Topic	No.	Item	Location where item is reported
**TITLE**			

**Title**	1	Identify the report as a systematic review.	Page 1

**ABSTRACT**			

**Abstract**	2	See the PRISMA 2020 for Abstracts checklist	

**INTRODUCTION**			

**Rationale**	3	Describe the rationale for the review in the context of existing knowledge.	Page 2
**Objectives**	4	Provide an explicit statement of the objective(s) or question(s) the review addresses.	Page 3

**METHODS**			

**Eligibility criteria**	5	Specify the inclusion and exclusion criteria for the review and how studies were grouped for the syntheses.	Page 4
**Information sources**	6	Specify all databases, registers, websites, organisations, reference lists and other sources searched or consulted to identify studies. Specify the date when each source was last searched or consulted.	Page 4
**Search strategy**	7	Present the full search strategies for all databases, registers and websites, including any filters and limits used.	Page 3–4
**Selection process**	8	Specify the methods used to decide whether a study met the inclusion criteria of the review, including how many reviewers screened each record and each report retrieved, whether they worked independently, and if applicable, details of automation tools used in the process.	Page 4
**Data collection process**	9	Specify the methods used to collect data from reports, including how many reviewers collected data from each report, whether they worked independently, any processes for obtaining or confirming data from study investigators, and if applicable, details of automation tools used in the process.	Page 4–5
**Data items**	10a	List and define all outcomes for which data were sought. Specify whether all results that were compatible with each outcome domain in each study were sought (e.g. for all measures, time points, analyses), and if not, the methods used to decide which results to collect.	Page 5
	10b	List and define all other variables for which data were sought (e.g. participant and intervention characteristics, funding sources). Describe any assumptions made about any missing or unclear information.	Page 5
**Study risk of bias assessment**	11	Specify the methods used to assess risk of bias in the included studies, including details of the tool(s) used, how many reviewers assessed each study and whether they worked independently, and if applicable, details of automation tools used in the process.	Page 5
**Effect measures**	12	Specify for each outcome the effect measure(s) (e.g. risk ratio, mean difference) used in the synthesis or presentation of results.	Page 6
**Synthesis methods**	13a	Describe the processes used to decide which studies were eligible for each synthesis (e.g. tabulating the study intervention characteristics and comparing against the planned groups for each synthesis (item 5)).	Page 6
	13b	Describe any methods required to prepare the data for presentation or synthesis, such as handling of missing summary statistics, or data conversions.	Page 5–6
	13c	Describe any methods used to tabulate or visually display results of individual studies and syntheses.	N/A
	13d	Describe any methods used to synthesize results and provide a rationale for the choice(s). If meta-analysis was performed, describe the model(s), method(s) to identify the presence and extent of statistical heterogeneity, and software package(s) used.	Page 6
	13e	Describe any methods used to explore possible causes of heterogeneity among study results (e.g. subgroup analysis, meta-regression).	Page 6
	13f	Describe any sensitivity analyses conducted to assess robustness of the synthesized results.	N/A
**Reporting bias assessment**	14	Describe any methods used to assess risk of bias due to missing results in a synthesis (arising from reporting biases).	Page 6
**Certainty assessment**	15	Describe any methods used to assess certainty (or confidence) in the body of evidence for an outcome.	Page 6

**RESULTS**			

**Study selection**	16a	Describe the results of the search and selection process, from the number of records identified in the search to the number of studies included in the review, ideally using a flow diagram.	Page 6
	16b	Cite studies that might appear to meet the inclusion criteria, but which were excluded, and explain why they were excluded.	Page 6
**Study characteristics**	17	Cite each included study and present its characteristics.	Page 6
**Risk of bias in studies**	18	Present assessments of risk of bias for each included study.	Page 6
**Results of individual studies**	19	For all outcomes, present, for each study: (a) summary statistics for each group (where appropriate) and (b) an effect estimate and its precision (e.g. confidence/credible interval), ideally using structured tables or plots.	Page 8
**Results of syntheses**	20a	For each synthesis, briefly summarise the characteristics and risk of bias among contributing studies.	Page 8–9
	20b	Present results of all statistical syntheses conducted. If meta-analysis was done, present for each the summary estimate and its precision (e.g. confidence/credible interval) and measures of statistical heterogeneity. If comparing groups, describe the direction of the effect.	Page 8–9
	20c	Present results of all investigations of possible causes of heterogeneity among study results.	Page 8–9
	20d	Present results of all sensitivity analyses conducted to assess the robustness of the synthesized results.	Page 8–9
**Reporting biases**	21	Present assessments of risk of bias due to missing results (arising from reporting biases) for each synthesis assessed.	Page 8–9
**Certainty of evidence**	22	Present assessments of certainty (or confidence) in the body of evidence for each outcome assessed.	Page 8–9

**DISCUSSION**			

**Discussion**	23a	Provide a general interpretation of the results in the context of other evidence.	Page 9–10
	23b	Discuss any limitations of the evidence included in the review.	Page 9–10
	23c	Discuss any limitations of the review processes used.	Page 9–10
	23d	Discuss implications of the results for practice, policy, and future research.	Page 9–10

**OTHER INFORMATION**			

**Registration and protocol**	24a	Provide registration information for the review, including register name and registration number, or state that the review was not registered.	N/a
	24b	Indicate where the review protocol can be accessed, or state that a protocol was not prepared.	N/A
	24c	Describe and explain any amendments to information provided at registration or in the protocol.	N/A
**Support**	25	Describe sources of financial or non-financial support for the review, and the role of the funders or sponsors in the review.	Page 11
**Competing interests**	26	Declare any competing interests of review authors.	Page 11
**Availability of data, code and other materials**	27	Report which of the following are publicly available and where they can be found: template data collection forms; data extracted from included studies; data used for all analyses; analytic code; any other materials used in the review.	N/A

### PRIMSA Abstract Checklist

[Table t3-04mjms3104_ra]

**Table t3-04mjms3104_ra:**

Topic	No.	Item	Reported?
**TITLE**			

**Title**	1	Identify the report as a systematic review.	Yes

**BACKGROUND**			

**Objectives**	2	Provide an explicit statement of the main objective(s) or question(s) the review addresses.	Yes

**METHODS**			

**Eligibility criteria**	3	Specify the inclusion and exclusion criteria for the review.	Yes
**Information sources**	4	Specify the information sources (e.g. databases, registers) used to identify studies and the date when each was last searched.	Yes
**Risk of bias**	5	Specify the methods used to assess risk of bias in the included studies.	Yes
**Synthesis of results**	6	Specify the methods used to present and synthesize results.	Yes

**RESULTS**			

**Included studies**	7	Give the total number of included studies and participants and summarise relevant characteristics of studies.	Yes
**Synthesis of results**	8	Present results for main outcomes, preferably indicating the number of included studies and participants for each. If meta-analysis was done, report the summary estimate and confidence/credible interval. If comparing groups, indicate the direction of the effect (i.e. which group is favoured).	Yes

**DISCUSSION**			

**Limitations of evidence**	9	Provide a brief summary of the limitations of the evidence included in the review (e.g. study risk of bias, inconsistency and imprecision).	Yes
**Interpretation**	10	Provide a general interpretation of the results and important implications.	Yes

**OTHER**			

**Funding**	11	Specify the primary source of funding for the review.	Yes
**Registration**	12	Provide the register name and registration number.	Yes

*Source:* Page MJ, McKenzie JE, Bossuyt PM, Boutron I, Hoffmann TC, Mulrow CD, et al. The PRISMA 2020 statement: an updated guideline for reporting systematic reviews. MetaArXiv. 2020, September 14. https://doi.org/10.31222/osf.io/v7gm2. For more information, visit: www.prisma-statement.org
